# Drug Discovery: Today and Tomorrow

**DOI:** 10.6026/97320630016001

**Published:** 2020-01-01

**Authors:** Darren R Flower

**Affiliations:** 1 Independent Interdisciplinary Consultant, Oxford, United Kingdom

## Abstract

Drug discovery continues to underperform relative to unmet medical need. Driven by profit not societal need, the search for new drugs is neither properly funded nor sufficiently systematic. Many innovative approaches are significantly underused yet extant methodology is replete with problems. In and of itself, technical innovation is unlikely to fulfill the potential of drug discovery if the supporting infrastructure remains unchanged.

## Background

The discovery of new medicines-traditionally drugs and vaccines, now expanded by a plethora of newer alternatives, including medical devices-is driven primarily by commercial interest rather than unmet medical need. Yet unmet need remains strong. Infectious disease and antibiotic resistance apart, there is, amongst others, still cancer and diseases of old-age, including dementia and other neurodegenerative maladies, with which to deal.

In recent decades, the clear failings of extant drug discovery have been mitigated by outrageous good fortune and the brilliance of individual research teams. Drug development costs remain high, driven by the expense of failure. Scarcely 1 in 12 drug candidates reach market, those entering clinical trials will typically possess activity and safety, yet still have a greater than 90% chance of failure, due to unexpected human side-effects or insufficient efficacy [1,2]. In this context, we will briefly elaborate some of the pressing issues with which drug discovery must deal now, and in the future.

## Animal Experimentation:

Traditional drug discovery has long relied on animal experiments to evaluate inter alia potency, selectivity, and toxicity. Ethical issues aside, poorly validated animal experiments often prove to be at best distracting and at worst misleading, providing data that is an unpredictably- inaccurate simulacrum of human disease [3]. For example, an adult male BALB/c mouse is 1/4500 the size of an adult human male yet perversely the presumption remains that despite such marked differences in phenotype and genotype, rodent models - probably the most pervasive pre-clinical and toxicology models - provide a seamless surrogate for human disease. Yet there are eminently viable and equally reliable alternatives: in vitro approaches, such as human organoids, as well as computational prediction.

## Clinical Trials:

The ultimate validation of a drug or vaccine’s efficacy and safety is the double- blind Randomized controlled trial (RCT), yet RCTs are also replete with problems. Apart from their expense and organizational complexity, a so-called clinical trial is seldom large enough or long enough nor properly stratified with respect to gender, age, and ethnicity, to properly evaluate a drug, particularly in respect of side-effects and adverse drug reactions, returning only partial, incomplete and often tendentious data about drug properties, necessitating long-term pharmacovigilance and potential product recalls. An RCT is meant to prevent future thalidomide disasters, yet Vioxx still reached market.

## Antibiotic Resistance:

To resist antibiotic resistance, new and effective antibiotics are desperately needed. Yet the response from the Pharmaceutical Industry has been sub-optimal. Partly for technical reasons, partly due to company strategies which see other areas of medical need as far more remunerative. Many strategies have been suggested to foster antibiotic development [4], but with mixed results. The solution to this dilemma is obvious, but deeply unpalatable: state-funded drug discovery. Only by bringing back scientifically- led drug discovery under public control, can we hope to address the full diversity of societal needs, in particular the discovery of new antibiotics, fully and completely.

## Vaccine Discovery:

Vaccination is the medical intervention par excellence: the wide deployment of vaccination during the last 220 years has significantly reduced (95-97%) in mortality and morbidity from a multitude of diseases: diphtheria, pneumonia, rubella, hepatitis B, tetanus, measles, mumps, and meningitis [5], as well as totally eradicating smallpox and almost eradicating polio. Modern licensed vaccines are whole organism-based or based on single proteins, as well as carbohydrate epitope-based vaccines. Single protein or so-called subunit vaccines are prime targets for vaccine design and reverse vaccinology. But the discovery and development of vaccines remains reliant on a variety of antiquated techniques and processes.

## Repurposing Drugs for New Indications:

Drug Repurposing is an area of translational biology that identifies novel therapeutically- useful indications for marketed drugs by identifying new, disease-relevant biological activities. Compounds that have been successfully evaluated for safety in Phase I clinical trials but proved unsuccessful for efficacy reasons in Phase II or Phase III trials may also be repurposed. Successful examples of such repositioning abound [6]. Most, maybe all, drugs have significant off-target activity, so drug repurposing has enormous and largely unexploited potential for the identification of safe, novel, well-tested medicines.

## Computational Drug Discovery: AI and beyond:

Artificial Intelligence (AI) is, with the greatest of respect to those involved, more hype than substance. AI is no different to what it was 5, 10, or 20 years ago. There has not been a technical step change, rather it results from greater investment; larger, faster computers and massive more data storage; more and better data; wider deployment of algorithms; but, of course, also a tsunami of specious hype. Perhaps, and due it must be said in part to the effect of that hype on Pharmaceutical management, AI has also created new opportunities for computational chemistry to finally have the effect that it could and should have had decades ago. Based on legacy data and properly designed in vitro models, the proper use of in silico methods can replace, reduce, and refine the use of expensive and time-consuming animal experiments and redundant clinical trials, and so help the discovery of new antibiotics, in its immuno-informatics manifestation help design novel vaccines [7-11], and power a repurposing revolution that uses automated protein-docking to systematically identified testable and already fully safety-tested candidate ligands for all human protein drug targets.

## Conclusion

Hitherto, drug discovery has progressed through a haphazard process of serendipity driven by the hunt for highly- remunerative low-hanging-fruit [12]. This is simply not sufficient to fully exploit the potential of drugs and vaccines as medical interventions. Science-led, complete and fully systematic, state-funded discovery of drugs and vaccines using reliable in vitro models and robust computational chemistry, validated by fewer yet larger and better-run double-blind clinical trials, looking for real advantages exhibited by tested drugs, rather than the marginal improvements evinced currently by many newly-approved NCEs. This in turn should lead to the definition of a so-called reduced pharmacopeia, comprising fewer yet safer drugs with many more properly understood activities and targeted medical indications, making global health-care at once more universal and more efficient and cost-effective.
